# Transcranial doppler assessment of preoperative cerebral blood flow velocity in cardiac surgery patients

**DOI:** 10.1007/s10877-025-01388-7

**Published:** 2025-12-12

**Authors:** Thomas Saller, Mahmoud Almaghrabi, Marcus Thudium, MHD Nedal Al Saqqa, Erich Kilger, Gerd Juchem

**Affiliations:** 1 Department of Anaesthesiology, University Hospital, LMU Munich, 81377 Munich, Germany; 2 Department of Anaesthesiology, University Hospital Bonn, 53127 Bonn, Germany; 3 Department of Statistics and Information Systems, Faculty of Economics, University of Damascus, Damascus, Syria; 4 Department of Cardiac Surgery, University Hospital, LMU Munich, 81377 Munich, Germany

**Keywords:** Postoperative delirium, Transcranial doppler, Blood flow velocity, Cerebral autoregulation, Cardiac surgery, Cardiopulmonary bypass

## Abstract

Postoperative delirium (POD) is a common and multifactorial complication following cardiac surgery, with cardiopulmonary bypass (CPB) playing a significant contributory role. Impaired cerebral autoregulation (CA) during CPB, particularly in older patients, may lead to cerebral hypo- or hyperperfusion. While several methods exist to assess CA and cerebral blood flow, many require specialized equipment not widely available. This prospective observational study aimed to investigate whether altered cerebral artery flow velocity, measured preoperatively by transcranial Doppler (TCD), is associated with the development of POD. We enrolled 41 patients undergoing elective cardiac surgery with CPB. Bilateral peak flow velocities of the middle cerebral arteries were measured preoperatively using TCD. The mean middle cerebral artery velocity (mMCAv_mean_) was calculated for each patient. POD occurred in 21 patients (51%). A lower mMCAv_mean_ was significantly associated with an increased risk of POD. Specifically, each 1 cm/s decrease in mMCAv_mean_ increased the likelihood of POD by 9.2% (odds ratio 0.908; 95% confidence interval: 0.840–0.981; *p =* 0.015). Reduced cerebral blood flow velocity during CPB, as measured by TCD, is associated with a higher risk of POD. These findings highlight the potential utility of intraoperative TCD monitoring for early identification of at-risk patients and support further research into TCD-guided preventive strategies in cardiac surgery.

## Introduction

Due to the ongoing demographic shift and an aging population, POD has become an increasingly important challenge in clinical practice, for which no definitive causal treatment has been established yet [1, 2]. POD is associated with elevated morbidity, mortality, and healthcare costs, thereby placing a substantial burden on healthcare systems worldwide [3, 4]. Delirium is a multifactorial disease [5]. The underlying pathology of this acute alteration of consciousness or cognitive state is complex and influenced by various risk factors. Both pre-existing medical conditions – such as advanced age [6], pre-existing neurocognitive deficits [6] and frailty [7], as well as perioperative factors – including cardiopulmonary bypass (CPB) use [3, 8], prolonged operative time [9], ICU stay duration [9], and total hospital stay duration [9] can substantially increase the risk of postoperative delirium.

Among patients for cardiac surgery, POD occurs in more than every second case [10], especially in open heart surgery and heart valve replacement [11, 12]. Although this population typically exhibits a higher prevalence of relevant risk factors, many risk prediction tools fail in cardiac surgery.

Several observational studies have shown that both hypoperfusion and hyperperfusion can lead to neurocognitive deficits [13–15]. Cerebral autoregulation (CA) refers to the brain’s intrinsic ability to maintain stable cerebral blood flow despite fluctuations in systemic blood pressure [16]. In the present study, we did not directly measure cerebral autoregulation; however, our analysis focuses on MCA flow velocity as a potential risk marker for POD. The concept of CA was first introduced by Lassen et al. in 1959, who also described the existence of a lower limit of mean arterial pressure (MAP) below which CA fails, a phenomenon now known as the lower limit of autoregulation (LLA) [17]. Subsequent studies by Drummond et al. have challenged the assumption that a MAP of 50 mmHg universally defines the LLA, emphasizing substantial interindividual variability in cerebral perfusion responses. However, hypoperfusion has been observed in some patients already at MAP values around 55 mmHg [18]. These findings have prompted ongoing research into more individualized approaches. Joshi et al. proposed that real-time perioperative monitoring of CA may offer a more accurate method for determining patient-specific LLA thresholds [19]. Building on this individualized approach, Hogue et al. conducted a study involving 460 patients and observed a significant reduction in POD incidence within the intervention group [20]. Similar results were reported by Brown et al. in a separate cohort of approximately 200 patients [21]. Nevertheless, not all studies have confirmed this association, highlighting the need for further investigation [22, 23]. Considering these inconsistent findings, our study aims to extend the understanding of POD risk by shifting focus from MAP thresholds to direct measurement of cerebral blood flow velocity. Specifically, we investigated the research question, whether decreased preoperative blood flow velocity in the MCA exhibits a risk for POD.

## Materials & methods

This prospective observational study was approved by the Ethics Committee of Ludwig-Maximilians-Universität Munich (IRB #22–0741). A priori sample size estimation was performed to ensure adequate statistical power. Based on a significance level of 0.05 and a power of 85%, a minimum of 18 patients per group was required. Participants were randomly selected in accordance with the ongoing surgical schedule at the Department of Cardiac Surgery. To ensure a homogeneous study population, patients aged ≥ 60 years were eligible for inclusion. All enrolled patients underwent cardiac surgery at the University Hospital of LMU Munich between October 2022 and April 2023.

### Inclusion criteria

Patients were 60 or more years of age, showed full ability to provide informed consent prior to participation, had no prior ischemic stroke, dementia, or other relevant neurological disorders, and had surgery planned with duration of 1 h or more under cardiopulmonary bypass (CPB), which served to exclude minor risk procedures. Patients were planned to stay postoperatively at least one night. Procedures included coronary artery bypass grafting (CABG), aortic or mitral valve replacement or reconstruction, and combination of these procedures.

### Exclusion criteria

included emergency cardiac procedures, inability to provide consent due to language barriers and/or relevant neurological disorders, known allergy to adhesive electrodes.

**Fig. 1:**
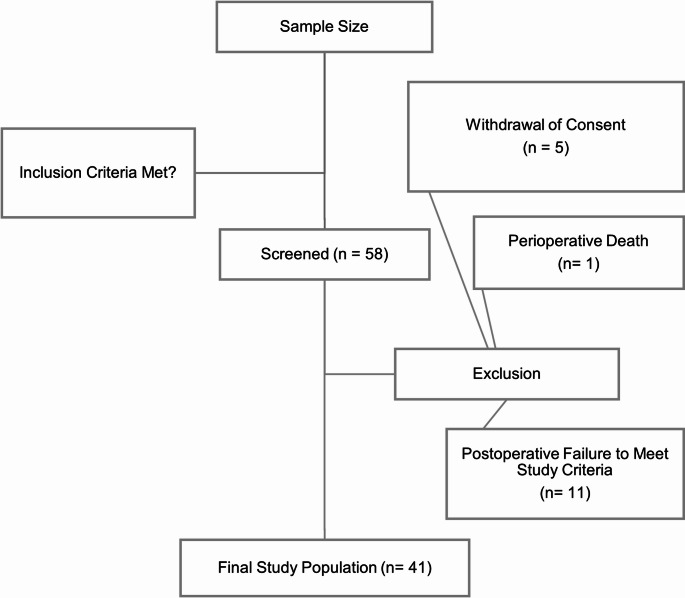
Study flow chart: process of study participants

Eligible patients were approached by a licensed physician and provided with standardized study information, including objectives, procedures, and potential benefits. Data collection commenced only after obtaining written informed consent. A total of 58 patients were initially screened. Of these, 17 patients were retrospectively excluded due to withdrawal of consent, intraoperative mortality, or subsequent failure to meet inclusion criteria, resulting in a final study sample of *n* = 41 patients. All participants received postoperative care according to the institutional standard protocol. Figure 1 illustrates the patient selection process for the study.

**Fig. 2:**
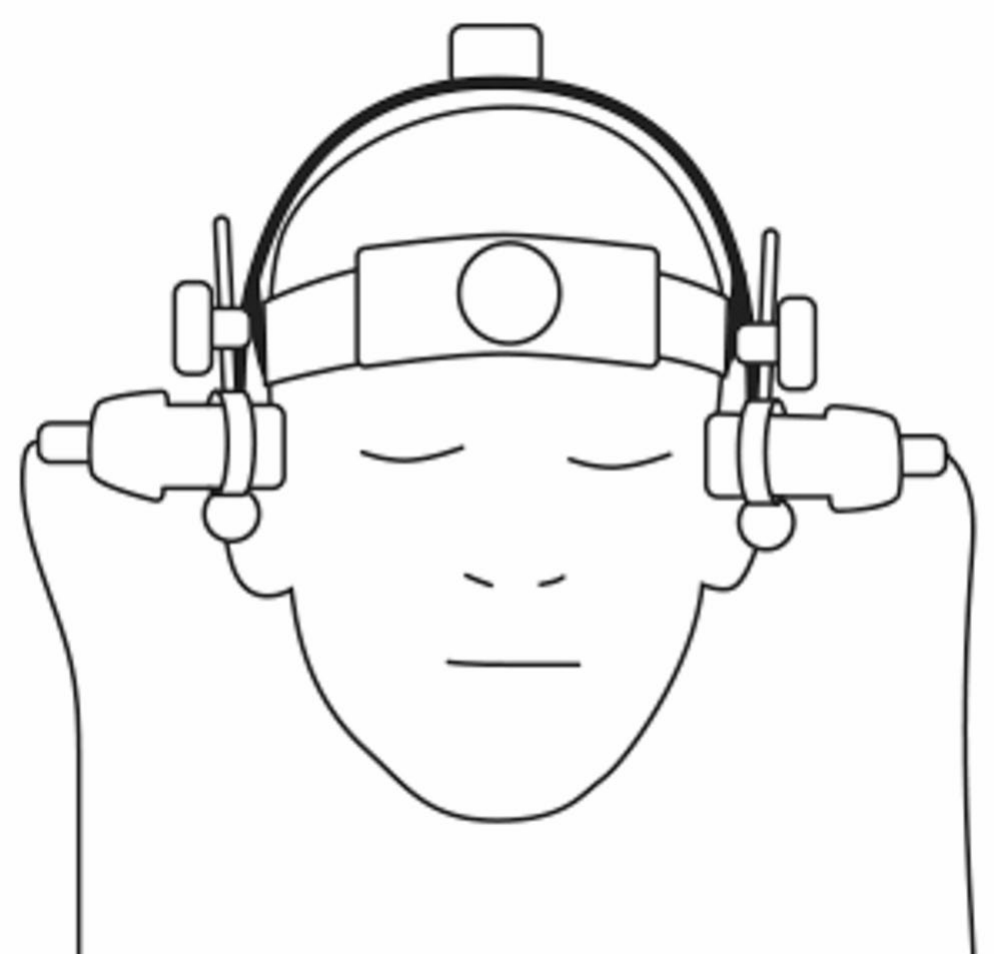
Schematic illustration of bilateral ultrasound probe placement for TCD [24]

Bilateral transcranial Doppler (TCD) measurements were performed (Fig. 2) using the DWL Doppler-Box (Compumedics Germany GmbH, Singen, Baden-Württemberg, Germany), equipped with DWL QL Routine Software including Doppler M-Mode. Two 2 MHz pulsed-wave (PW) transducers were bilaterally positioned to insonate the middle cerebral arteries (MCAs) [24]. In cases where no signal could be performed, ultrasound guidance with colour doppler was used to identify the cerebral vasculature. The transducers were secured with a head-mounted frame to maintain stable positioning during the examination. The procedure was non-invasive, and no complications were expected. However, in rare cases, patients with highly sensitive skin could experience allergic reactions to either the Doppler transducers or the adhesive materials used for probe fixation. The Doppler-Box system was operated via a Microsoft Windows-compatible interface, allowing real-time visualization of flow parameters.

Prior to study initiation, the TCD device underwent technical validation to ensure signal quality and measurement reliability. Investigators completed a three-month training program before patient enrollment to minimize operator-related variability.

To evaluate the study hypothesis, preoperative blood flow velocity measurements of the bilateral middle cerebral arteries (MCAs) were performed on the surgical ward with awake patients, after their admission on the day before surgery. Attempts to perform intraoperative measurements were technically unsuccessful in this pilot cohort. Three parameters were specifically analyzed: vessel depth, peak velocity (MCAv_peak_), and mean flow velocity (MCAv_mean_). The arithmetic mean of the MCAv_mean_ values from both hemispheres (mMCAv_mean_) was then calculated and used as an individual reference value. This parameter was subsequently assessed for its potential association with the development of POD. In addition, baseline patient characteristics, underlying medical conditions, types of surgical procedures performed, and relevant perioperative and postoperative times were systematically recorded. Frailty was assessed using the Edmonton Frail Scale and the Fried Scale [25, 26].

No sedative premedication was administered preoperatively according to institutional standards. Neuropsychological assessments were conducted both preoperatively and postoperatively to evaluate cognitive function and attentional capacity. The Mini-Cog test was administered preoperatively to assess cognitive status [27]. Postoperatively, neuropsychological testing was repeated daily over the first five postoperative days to monitor the development of POD by trained study personnel. The 3D-CAM test (3 min Diagnostic Interview for CAM-defined delirium) was used on the surgical ward to assess delirium [28], while either the CAM-ICU or the Nursing Delirium Screening Scale (Nu-DESC) was applied in the ICU setting once per shift [29, 30]. A positive neuropsychological test result within the first five postoperative days was considered as the presence of POD [31].

To ensure patient confidentiality, all collected personal data were anonymized and stored in an Excel file on a password-protected computer within the clinical setting. Access to this data was restricted to authorized study personnel bound by confidentiality agreements. Statistical analyses were conducted using IBM SPSS Statistics software. Descriptive statistics were initially generated to summarize the data. Subsequently, logistic regression analysis was applied to variables with significant differences (*p* < 0.05) to identify correlations and potential risk factors associated with the development of POD.

Normality tests were performed for each variable, and statistical methods were selected accordingly. Parametric measures were applied when the assumption of normality was met, and non-parametric tests when it was not. Significant group differences were observed particularly for weight, CPB time, incision-to-suture duration, ICU Stay duration and MCAv_mean_ values.

## Results

**Fig. 3:**
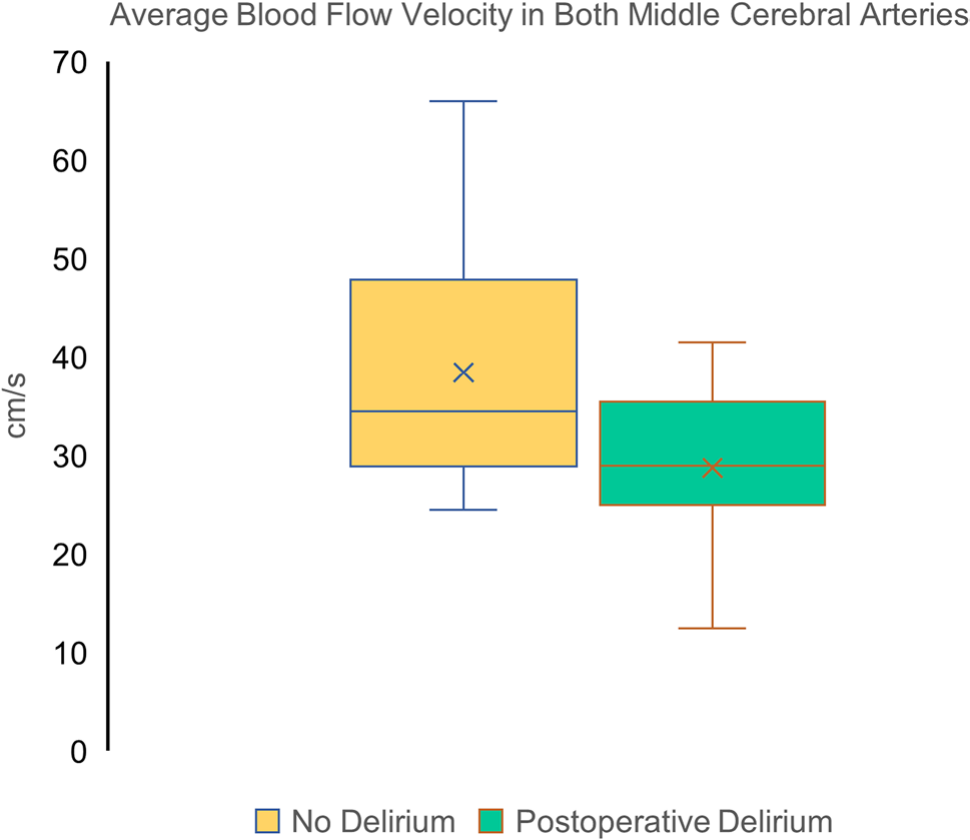
Distribution of mMCAv_mean_ by postoperative delirium outcome

A total of 41 patients were included in the study cohort. 21 patients (51%) developed delirium. There was a predominance of male patients (68.3% male vs. 31.7% female). Baseline characteristics and perioperative variables are summarized in Table 1, and transcranial Doppler measurements in Table 2. Figure 3 illustrates the distribution of mMCAv_mean_, highlighting that patients with POD had consistently lower values compared with those without POD.

**Table 1:** Demographic, medical, surgical, and postoperative characteristics of patients with POD (*n* = 21) and without POD (*n* = 20)

Variable	No Delirium *n* = 20	Delirium *n* = 21	*P*-value
Age (years)	69.4 ± 5.1	71 ± 5.3	0.348
Gender			
Male	16 (80%)	12 (57.1%)	0.116
Female	4 (20%)	9 (42.9%)	
Height (cm)	175.1 ± 8.6	171.5 ± 8.1	0.185
Weight (kg)	90.8 ± 20	77 ± 18	0.032*
BMI	29.5 ± 6.6	26 ± 4.6	0.052
Weight 1 year ago (kg)	93 ± 21	78 ± 17.4	0.018*
Edmonton Frail Scale Not frail (score = 0) Pre-frail/frail (score ≥ 1)			
	13 (65%)	11 (52.4%)	0.412
	7 (35%)	10 (47.6%)	
Fried frailty scale			
Not frail (score = 0)	9 (45%)	9 (39.1%)	0.890
Pre-frail/frail (score ≥ 1)	11 (55%)	12 (60.9%)	
Type of surgery			
Valve replacement	9 (45%)	10 (47.6%)	0.488
Aortic procedures	5 (25%)	5 (23.8%)	
Combined procedures	4 (20%)	6 (28.6%)	
Other	2 (10%)	0 (0%)	
Incision-to-suture time (min)	272 ± 91	364 ± 92	0.003*
Bypass time (min)	122 ± 68	178 ± 70	0.014*
Reperfusion time (min)	36 ± 27	59 ± 12	0.081
Circulatory arrest time (min)	19 ± 17	26 ± 4	0.327
Temperature minimum (°C)	22,6 ± 0.3	22,9 ± 0.4	0.160
Aortic clamp time (min)	82 ± 50	106 ± 43	0.101
ICU stay duration (hours)	44.7 [22–96]	90 [44–168]	0.050*
IMC stay duration (hours)	73.7 [48–132]	84 [46–145]	0.770
Postoperative stay duration (days)	13 ± 5	18 ± 9	0.023*
Total Hospital stay duration (days)	15 ± 6	23 ± 10	0.006*

Data are presented as mean ± SD or median [IQR], as appropriate. (*) Statistically significant difference (*p* < 0.05)

**Table 2:** Transcranial doppler measurement of MCA blood flow velocity

Variable	No Delirium *n* = 20	Delirium *n* = 21	*P*-value
Right MCA depth (mm)	47.1 ± 6.9	52 ± 12.2	0.081
Left MCA depth (mm)	45 [40–50]	56 [38–65]	0.416
Right MCA peak velocity (cm/s)	56 [49–77]	57 [48–69]	0.811
Left MCA peak velocity (cm/s)	65 ± 19	58 ± 15	0.154
Right MCA mean velocity (cm/s)	33 [28–47]	29 [27–38]	0.266
Left MCA mean velocity (cm/s)	38 ± 12.3	29 ± 6.7	0.007*
Average MCA mean velocity (cm/s)	37 [30–47]	30 [27–36]	0.020*

Data are presented as mean ± SD or median [IQR], as appropriate. (*) Statistically significant difference (*p* < 0.05)

Frailty assessment revealed no statistically significant differences, although patients who developed POD had lower body weight and greater weight loss over the preceding year. Regarding surgical factors, the distribution of procedure types (aortic, valve, or combined) did not show any significant difference between groups. Although valve procedures are generally considered a risk factor for POD, in our cohort procedure type was not significantly associated with delirium. By contrast, operative parameters showed that patients developing POD had significantly longer incision-to-suture times and CPB times with regression analyses confirming these associations (incision-to-suture: odds ratio OR = 1.012 [95% CI 1.003–1.022], *p =* 0.009; CPB time: OR = 1.013 [1.002–1.024], *p =* 0.025). Median ICU stay was 73.7–84 h and tended to be longer in POD patients, although regression did not confirm this association (OR = 1.007 [0.997–1.017], *p =* 0.194). At our institution, no formal fast-track protocol is in place; patients remain in the ICU as long as clinically indicated. Both postoperative (OR = 1.129 [1.006–1.268], *p =* 0.039) and total hospital (OR = 1.142 [1.024–1.274], *p =* 0.017) stay were significantly longer in the POD group.

In addition to these perioperative characteristics, we evaluated the relationship between preoperative cerebral blood flow velocity and POD. Following the evaluation of the measured data, a univariate logistic regression analysis was performed. Right MCAv_mean_ remained non-significant (OR = 0.958 [0.903–1.016], *p =* 0.153), while left MCAv_mean_ was significantly associated with POD (OR = 0.905 [0.833–0.982], *p =* 0.017). Likewise, mMCAv_mean_ demonstrated a significant association (OR = 0.908 [0.840–0.981], *p =* 0.015). In multivariate logistic regression, adjusting for depth and peak flow both right MCAv_mean_ (OR = 0.837 [0.713–0.983], *p =* 0.030) and left MCAv_mean_ (OR = 0.829 [0.718–0.956], *p =* 0.010) were significant.

**Fig. 4:**
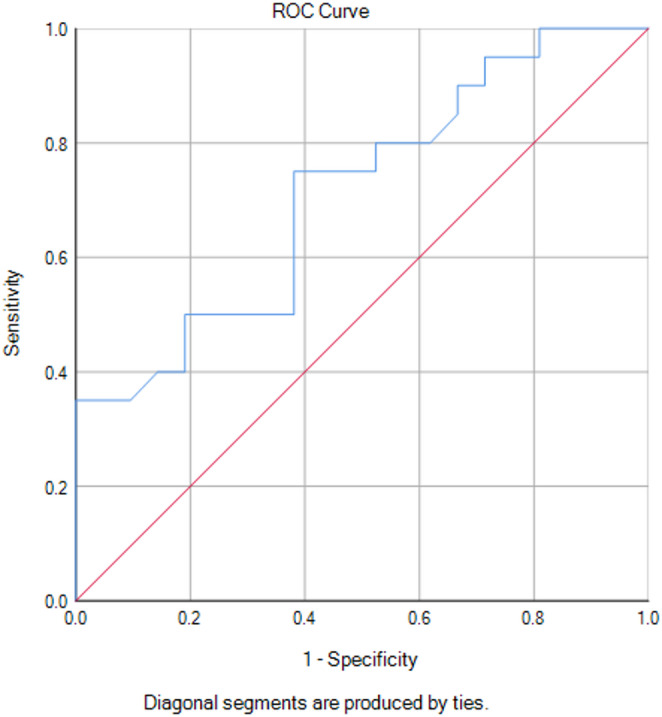
The ROC curve for mMCAv_mean_

Furthermore, multivariate regression models were conducted to evaluate the robustness of the association between mMCAv_mean_ and POD after adjusting for potential confounders. Adjustment for frailty measures confirmed the persistence of a significant association, both for the Fried Frailty Score (OR = 0.886 [0.807–0.972], *p =* 0.010) and the Edmonton Frail Scale (EFS) (OR = 0.905 [0.837–0.979], *p =* 0.013). Similarly, including perioperative variables such as incision-to-suture time and CPB time in the model, mMCAv_mean_ remained significantly associated with POD (OR = 0.896 [0.812–0.988], *p =* 0.028). Further adjustment for postoperative factors, including ICU stay and postoperative duration, also confirmed the association (OR = 0.198 [0.197–0.200], *p =* 0.010). Finally, a receiver operating characteristic (ROC) curve analysis (Fig. 4) was conducted, demonstrating a sensitivity of 75% and specificity of 61.9% at a threshold of 29.75 cm/s, with sensitivity improving to 95% at 25.25 cm/s.

## Discussion

### Summary of findings

The primary focus of our investigation was to examine the correlation between preoperative cerebral blood flow velocity and the incidence of POD. In our study of 41 patients undergoing cardiac surgery, a decreased mean velocity of the middle cerebral arteries (mMCAv_mean_), measured preoperatively by transcranial doppler sonography, was found to be significantly associated with an increased risk of POD, and this association remained robust across all adjusted models.

### Interpretation of key results

Intraindividual variations in blood flow were observed, These may be attributed to measurement errors, anatomical variations [32, 33], or pathological conditions [34]. The left MCA consistently showed significant differences related to POD occurrence, whereas the right MCA exhibited a similar but non-significant trend. A multiple regression model, including all TCD parameters for each hemisphere, revealed that both right and left blood flows were significant, suggesting that right MCAv_mean_ assessment may be more precise when adjusting for vessel depth and peak flow. In contrast, left MCAv_mean_ consistently demonstrated significant group differences, indicating reduced variability and potential as an independent predictor for POD.

This observed hemispheric asymmetry in predictive consistency may reflect underlying structural or regulatory differences between cerebral hemispheres. Additionally, there may be lateralized differences in cerebrovascular autoregulation or metabolic demand that contribute to the more consistent relationship between left MCAv_mean_ and POD. To minimize measurement errors and side-specific differences, mMCAv_mean_ was selected as the key study variable.

Box plot analysis (Fig. 3) showed that patients who developed POD had a significantly lower mMCAv_mean_ than those without POD (Delirium: 30 [IQR 27–36] vs. No Delirium: 37 [IQR 30–47]; *p =* 0.020), indicating a strong association between preoperatively reduced cerebral blood flow velocity and POD occurrence. The observed decrease in cerebral blood flow velocity suggests impaired cerebral perfusion and disrupted autoregulation, increasing vulnerability to POD. Simple logistic regression confirmed this association, with an odds ratio of 0.908 [0.840–0.981], *p =* 0.015, indicating that a 1 cm/s decrease in mean blood flow increases the likelihood of POD by 9.2%.

Our hypothesis that lower blood flow velocity correlates with increased POD risk is supported by findings at a ROC threshold of 25.25 cm/s, where sensitivity increased to 95%. The associated disadvantage, however, lies in the markedly reduced specificity of 28.6%, increasing the rate of false positives. This trade-off is accepted in favor of early identification of at-risk patients, which remains a clinical priority. To further validate these findings, multiple logistic regression models were applied, adjusting for frailty, perioperative variables, and postoperative variables. In all models, mean blood flow remained a significant predictor, confirming a robust and reliable association between reduced cerebral perfusion and POD. The origin of this association remains unknown. Increased MCAv suggests a narrower vessel as it is observed with cerebro-arterial macroangiopathy. Conversely, reduced MCAv is associated with increased cardiovascular risk [35], and decreased total cerebral blood flow (CBF) has been linked to higher mortality [36].

### Integration with existing literature

Our findings align with prior studies suggesting that impaired cerebral perfusion is a key contributor to neurocognitive decline in elderly surgical patients [13–15, 35, 36]. In a recent study, optimizing the lower limit of autoregulation by increased CPB did not alter LLA [37]. However, while much of the existing literature focuses on MAP-based autoregulation thresholds or intraoperatively measured blood velocity, our results suggest that lower preoperative MCAv_mean_ may serve as an early indicator of increased vulnerability for POD. This perspective complements current evidence and contributes to a growing body of evidence supporting the value of individualized cerebral hemodynamic assessment in perioperative risk stratification. To our knowledge, current literature lacks direct comparisons between hemispheric MCAv_mean_ patterns in relation to POD. This makes our findings particularly novel and underscores the need to consider lateralization in cerebral hemodynamics. It also presents a valuable opportunity for future research to explore the mechanisms underlying hemispheric asymmetry and its implications for personalized risk assessment.

### Methodological strengths and limitations

Our study benefits from standardized bilateral TCD measurements, strict inclusion criteria, and comprehensive statistical adjustment. However, several limitations must be acknowledged: TCD is operator dependent, the small sample size limits generalizability, and the observational nature of this study precludes a comprehensive evaluation of the impact of individualized hemodynamic management on POD incidence following CPB. Additionally, although included in the analysis, gender and age differences may be an unknown risk of bias to our results that merits further validation.

Furthermore, our main research question and hypothesis focused on whether preoperative cerebral blood flow velocity measured by TCD is associated with POD. Other established risk factors such as atrial fibrillation, carotid artery stenosis, smoking, alcohol use, or renal insufficiency were therefore not systematically captured, as this extends beyond the core trajectory of the present study. These factors are well recognized in the literature as important contributors to POD and should be addressed in future investigations.

Moreover, our data do not investigate the perioperative changes in cerebral hemodynamics. The evident loss of autoregulation during CPB is the most likely origin of hypoperfusion or hyperperfusion associated with worse cognitive outcome. Preoperative assessment may indicate patients at-risk. Nevertheless, additional intra- and postoperative measurements would provide valuable insight into the dynamic relationship between CPB, cerebral perfusion, and POD. It is also conceivable that patients with lower preoperative velocities may be particularly prone to relative hyperperfusion once non-pulsatile CPB flow is initiated, a mechanism that could contribute to POD. Therefore, prospective studies examining this association will be performed.

Despite these limitations, the consistent association between preoperative MCAv_mean_ and POD across multiple adjusted models underlines the robustness of our findings and supports the potential utility of preoperative TCD as a non-invasive screening tool for risk stratification.

### Future research directions

mMCAv_mean_ during rest provides valuable information for establishing a baseline. However, incorporating MCAv_mean_ during physical activity may offer a more comprehensive assessment of cerebrovascular responsiveness to physiological demand [38] and could be examined in future studies. Furthermore, intraoperative CPB flow could be adjusted to match each patient’s preoperatively measured MCAv threshold, thereby preventing hypoperfusion and hyperperfusion. TCD ultrasound represents one of the most effective non-invasive methods available for monitoring cerebral hemodynamics and thus potentially preventing POD. In the ongoing discussion about the use of pulsatile flow during cardiopulmonary bypass, TCD-controlled MCAv-assessment before, during and after CPB may be a secondary endpoint worthwhile. As cardiac surgery under CPB is one of the most important precipitating factors for delirium, MCAv assessment may be one step to early identify high-risk patients, and support timely implementation of preventive strategies, both preoperatively and intraoperatively. Nevertheless, further research is needed to clarify the effect of patient-adapted hemodynamic management on POD incidence. Additionally, systematic training of clinical personnel in TCD-application is essential to minimize operator-related errors and ensure reliable measurements.

### Summary of discussion

In summary, our findings indicate that reduced preoperative MCAv_mean_ may be a new risk factor for delirium in cardiac surgery. These results underscore the potential of TCD as a non-invasive, accessible screening tool in the preoperative assessment of neurocognitive risk. Further prospective studies are needed to validate these findings and to evaluate whether individualized perfusion strategies based on TCD data can reduce the incidence of POD.

## Conclusion

Delirium is the most common complication following cardiac surgery. Reduced average blood flow velocity of both middle cerebral arteries (mMCAv_mean_) as determined by transcranial Doppler sonography was shown to be associated with delirium after cardiac surgery. Patients with low blood flow velocity values preoperatively showed significantly higher risk of developing POD, indicating that preoperative TCD-values may represent an indicator for POD. We observed that the likelihood of POD to increase significantly by 9.2% for each 1 cm/s decrease in the (mMCAv_mean_). Further research is needed to determine whether modifying CPB parameters based on preoperative TCD measurements can help reduce the incidence of delirium.

## Data Availability

The data that support the findings of this study are available upon reasonable request. The data are not publicly available due to the privacy and confidentiality of the research institution mandate.

## References

[1] Wilson JE, Mart MF, Cunningham C, Shehabi Y, Girard TD, MacLullich AMJ, et al. Delirium. Nat Rev Dis Primers. 2020;6:90. 10.1038/s41572-020-00223-4. 33184265 10.1038/s41572-020-00223-4 PMC9012267

[2] Oh S-T, Park JY. Postoperative delirium. Korean J Anesthesiol. 2019;72:4–12. 10.4097/kja.d.18.00073.1. 30139213 10.4097/kja.d.18.00073.1 PMC6369344

[3] Ouimet S, Kavanagh BP, Gottfried SB, Skrobik Y. Incidence, risk factors and consequences of ICU delirium. Intensive Care Med. 2007;33:66–73. 10.1007/s00134-006-0399-8. 17102966 10.1007/s00134-006-0399-8

[4] Vasilevskis EE, Ely EW, Speroff T, Pun BT, Boehm L, Dittus RS. Reducing iatrogenic risks. Chest. 2010;138:1224–33. 10.1378/chest.10-0466. 21051398 10.1378/chest.10-0466 PMC4694109

[5] Inouye SK, Charpentier PA. Precipitating factors for delirium in hospitalized elderly persons. Predictive model and interrelationship with baseline vulnerability. JAMA. 1996;275:852–7. 8596223

[6] Gosselt AN, Slooter AJ, Boere PR, Zaal IJ. Risk factors for delirium after on-pump cardiac surgery: a systematic review. Crit Care. 2015;19:346. 10.1186/s13054-015-1060-0. 26395253 10.1186/s13054-015-1060-0 PMC4579578

[7] Gracie TJ, Caufield-Noll C, Wang N-Y, Sieber FE. The association of preoperative frailty and postoperative delirium: a meta-analysis. Anesth Analg. 2021;133:314–23. 10.1213/ANE.0000000000005609. 34257192 10.1213/ANE.0000000000005609 PMC8289124

[8] Caldas JR, Haunton VJ, Panerai RB, Hajjar LA, Robinson TG. Cerebral autoregulation in cardiopulmonary bypass surgery: a systematic review. Interact Cardiovasc Thorac Surg. 2018;26:494–503. 10.1093/icvts/ivx357. 29155938 10.1093/icvts/ivx357

[9] Galyfos GC, Geropapas GE, Sianou A, Sigala F, Filis K. Risk factors for postoperative delirium in patients undergoing vascular surgery. J Vasc Surg. 2017;66:937–46. 10.1016/j.jvs.2017.03.439. 28583731 10.1016/j.jvs.2017.03.439

[10] Rudolph JL, Jones RN, Levkoff SE, Rockett C, Inouye SK, Sellke FW, et al. Derivation and validation of a preoperative prediction rule for delirium after cardiac surgery. Circulation. 2009;119:229–36. 10.1161/CIRCULATIONAHA.108.795260. 19118253 10.1161/CIRCULATIONAHA.108.795260 PMC2735244

[11] Shi SM, Sung M, Afilalo J, Lipsitz LA, Kim CA, Popma JJ, et al. Delirium incidence and functional outcomes after transcatheter and surgical aortic valve replacement. J Am Geriatr Soc. 2019;67:1393–401. 10.1111/jgs.15867. 30882905 10.1111/jgs.15867 PMC6612597

[12] Humbert M, Büla CJ, Muller O, Krief H, Monney P. Delirium in older patients undergoing aortic valve replacement: incidence, predictors, and cognitive prognosis. BMC Geriatr. 2021;21:153. 10.1186/s12877-021-02100-5. 33653285 10.1186/s12877-021-02100-5 PMC7927377

[13] Glumac S, Kardum G, Karanovic N. Postoperative cognitive decline after cardiac surgery: a narrative review of current knowledge in 2019. Med Sci Monit. 2019;25:3262–70. 10.12659/MSM.914435. 31048667 10.12659/MSM.914435 PMC6511113

[14] Guarracino F. Cerebral monitoring during cardiovascular surgery. Curr Opin Anaesthesiol. 2008;21:50–4. 10.1097/ACO.0b013e3282f3f499. 18195610 10.1097/ACO.0b013e3282f3f499

[15] Hori D, Brown C, Ono M, Rappold T, Sieber F, Gottschalk A, et al. Arterial pressure above the upper cerebral autoregulation limit during cardiopulmonary bypass is associated with postoperative delirium. Br J Anaesth. 2014;113:1009–17. 10.1093/bja/aeu319. 25256545 10.1093/bja/aeu319 PMC4235573

[16] Paulson OB, Strandgaard S, Edvinsson L. Cerebral autoregulation. Cerebrovasc Brain Metab Rev. 1990;2:161–92. 2201348

[17] Lassen NA. Cerebral blood flow and oxygen consumption in man. Physiol Rev. 1959;39:183–238. 10.1152/physrev.1959.39.2.183. 13645234 10.1152/physrev.1959.39.2.183

[18] Drummond JC. The lower limit of autoregulation: time to revise our thinking? Anesthesiology. 1997;86:1431–3. 10.1097/00000542-199706000-00034. 9197320 10.1097/00000542-199706000-00034

[19] Joshi B, Ono M, Brown C, Brady K, Easley RB, Yenokyan G, et al. Predicting the limits of cerebral autoregulation during cardiopulmonary bypass. Anesth Analg. 2012;114:503–10. 10.1213/ANE.0b013e31823d292a. 22104067 10.1213/ANE.0b013e31823d292a PMC3288415

[20] Hogue CW, Brown CH, Hori D, Ono M, Nomura Y, Balmert LC, et al. Personalized blood pressure management during cardiac surgery with cerebral autoregulation monitoring: a randomized trial. Semin Thorac Cardiovasc Surg. 2021;33:429–38. 10.1053/j.semtcvs.2020.09.032. 33186735 10.1053/j.semtcvs.2020.09.032

[21] Brown CH, Neufeld KJ, Tian J, Probert J, LaFlam A, Max L, et al. Effect of targeting mean arterial pressure during cardiopulmonary bypass by monitoring cerebral autoregulation on postsurgical delirium among older patients: a nested randomized clinical trial. JAMA Surg. 2019;154:819–26. 10.1001/jamasurg.2019.1163. 31116358 10.1001/jamasurg.2019.1163 PMC6537779

[22] Wesselink EM, Kappen TH, van Klei WA, Dieleman JM, van Dijk D, Slooter AJC. Intraoperative hypotension and delirium after on-pump cardiac surgery. Br J Anaesth. 2015;115:427–33. 10.1093/bja/aev256. 26209856 10.1093/bja/aev256 PMC4635646

[23] Langer T, Santini A, Zadek F, Chiodi M, Pugni P, Cordolcini V, et al. Intraoperative hypotension is not associated with postoperative cognitive dysfunction in elderly patients undergoing general anesthesia for surgery: results of a randomized controlled pilot trial. J Clin Anesth. 2019;52:111–8. 10.1016/j.jclinane.2018.09.021. 30243062 10.1016/j.jclinane.2018.09.021

[24] Compumedics Germany GmbH. Doppler-BoxX – Transkranielle Dopplersonographie: Hochleistungsfähiges TCD-System zur Untersuchung der zerebralen Hämodynamik. 9/1/2023. https://www.dwl.de/produkte/dbx-serie/dbx/. Accessed 15 Jun 2025.

[25] Rolfson DB, Majumdar SR, Tsuyuki RT, Tahir A, Rockwood K. Validity and reliability of the Edmonton frail scale. Age Ageing. 2006;35:526–9. 10.1093/ageing/afl041. 16757522 10.1093/ageing/afl041 PMC5955195

[26] Fried LP, Tangen CM, Walston J, Newman AB, Hirsch C, Gottdiener J, et al. Frailty in older adults: evidence for a phenotype. J Gerontol Biol Sci Med Sci. 2001;56:M146–56. 10.1093/gerona/56.3.m146. 10.1093/gerona/56.3.m146 11253156

[27] Fage BA, Chan CCH, Gill SS, Noel-Storr AH, Herrmann N, Smailagic N, et al. Mini-Cog for the diagnosis of Alzheimer’s disease dementia and other dementias within a community setting. Cochrane Database Syst Rev. 2015;CD010860. 10.1002/14651858.CD010860.pub2. 10.1002/14651858.CD010860.pub2 25922857

[28] Marcantonio ER, Ngo LH, O’Connor M, Jones RN, Crane PK, Metzger ED, Inouye SK. 3D-CAM: derivation and validation of a 3-minute diagnostic interview for CAM-defined delirium: a cross-sectional diagnostic test study. Ann Intern Med. 2014;161:554–61. 10.7326/M14-0865. 25329203 10.7326/M14-0865 PMC4319978

[29] Lütz A, Radtke FM, Franck M, Seeling M, Gaudreau J-D, Kleinwächter R, et al. Die nursing delirium screening scale (Nu-DESC) - Richtlinienkonforme ubersetzung für Den deutschsprachigen Raum. [The nursing delirium screening scale (NU-DESC)]. Anasthesiol Intensivmed Notfallmed Schmerzther. 2008;43:98–102. 10.1055/s-2008-1060551. 18293243 10.1055/s-2008-1060551

[30] Kotfis K, Marra A, Ely EW. ICU delirium - a diagnostic and therapeutic challenge in the intensive care unit. Anaesthesiol Intensive Ther. 2018;50:160–7. 10.5603/AIT.a2018.0011. 29882581 10.5603/AIT.a2018.0011

[31] Evered L, Silbert B, Knopman DS, Scott DA, DeKosky ST, Rasmussen LS, et al. Recommendations for the nomenclature of cognitive change associated with anaesthesia and surgery-2018. Br J Anaesth. 2018;121:1005–12. 10.1016/j.bja.2017.11.087. 30336844 10.1016/j.bja.2017.11.087 PMC7069032

[32] LASSEN NA, Ingvar DH, Skinhøj E. Brain function and blood flow. Sci Am. 1978;239:62–71. 10.1038/scientificamerican1078-62. 705327 10.1038/scientificamerican1078-62

[33] Naidich TP, Castillo M, Cha S, Smirniotopoulos JG. Imaging of the Brain E-Book: Expert Radiology Series. Elsevier Health Sciences; 2012.

[34] Hui C, Tadi P, Khan Suheb MZ, Patti L. StatPearls: Ischemic Stroke. Treasure Island (FL); 2025.

[35] Pase MP, Grima NA, Stough CK, Scholey A, Pipingas A. Cardiovascular disease risk and cerebral blood flow velocity. Stroke. 2012;43:2803–5. 10.1161/STROKEAHA.112.666727. 22879097 10.1161/STROKEAHA.112.666727

[36] Sabayan B, van der Grond J, Westendorp RG, Jukema JW, Ford I, Buckley BM, et al. Total cerebral blood flow and mortality in old age: a 12-year follow-up study. Neurology. 2013;81:1922–9. 10.1212/01.wnl.0000436618.48402.da. 24174588 10.1212/01.wnl.0000436618.48402.da PMC3843381

[37] Desebbe O, Berna A, Joosten A, Raphael D, Malapert G, Rolo D, et al. Impact of cardiopulmonary bypass flow on the lower limit of cerebral autoregulation during cardiac surgery: a randomized cross-over pilot study. J Clin Monit Comput. 2025;39:571–80. 10.1007/s10877-025-01290-2. 40220213 10.1007/s10877-025-01290-2

[38] Perdomo SJ, Ward J, Liu Y, Vidoni ED, Sisante JF, Kirkendoll K, et al. Cardiovascular disease risk is associated with middle cerebral artery blood flow velocity in older adults. Cardiopulm Phys Ther J. 2020;31:38–46. 10.1097/cpt.0000000000000110. 33100924 10.1097/cpt.0000000000000110 PMC7580865

